# Modified Metabolic Syndrome Criteria Considering Cardio-Ankle Vascular Index (CAVI) and A Body Shape Index (ABSI): Implications for Kidney Risk

**DOI:** 10.31083/RCM26583

**Published:** 2025-03-18

**Authors:** Daiji Nagayama, Yasuhiro Watanabe, Kohji Shirai, Masahiro Ohira, Atsuhito Saiki

**Affiliations:** ^1^Department of Internal Medicine, Nagayama Clinic, 323-0032 Oyama, Tochigi, Japan; ^2^Center of Diabetes, Endocrinology and Metabolism, Toho University, Sakura Medical Center, 285-0841 Sakura, Chiba, Japan; ^3^Division of Diabetes, Metabolism and Endocrinology, Toho University, Ohashi Medical Center, Meguro-Ku, 153-8515 Tokyo, Japan; ^4^Department of Internal Medicine, Mihama Hospital, 261-0013 Chiba, Chiba, Japan

**Keywords:** metabolic syndrome, waist circumference, a body shape index, cardio-ankle vascular index, vascular function, kidney function decline

## Abstract

Waist circumference (WC), an abdominal obesity index in the current metabolic syndrome (MetS) criteria, may not adequately reflect visceral fat accumulation. This brief review aims to examine the clinical significance of utilizing a body shape index (ABSI), a novel abdominal obesity index, to modify the MetS criteria, considering the predictive ability for vascular dysfunction indicated by the cardio-ankle vascular index (CAVI), as well as kidney function decline. First, the relationship of CAVI with kidney function is presented. Next, whether modification of the MetS diagnostic criteria by replacing the current high waist circumference (WC-MetS) with high ABSI (ABSI-MetS) improves the predictive ability for vascular and kidney dysfunction is discussed. Although limited to Asian populations, several cross-sectional and longitudinal studies support the relationship of CAVI with kidney function. Increased CAVI is associated with kidney function decline, and the CAVI cutoff for kidney outcomes is considered to be 8–9. In urban residents who underwent health screening, an increase in ABSI, but not body mass index (BMI) or WC, was associated with increased CAVI, suggesting that ABSI reflects vasoinjurious body composition. In several cross-sectional studies, ABSI-MetS was superior to WC-MetS in identifying individuals with increased CAVI. Furthermore, the predictive ability of ABSI-MetS in assessing kidney function decline was enhanced only in individuals with MetS, as determined in a longitudinal analysis. Using WC as a major criterion for MetS diagnosis may not adequately identify individuals at risk of vascular dysfunction and kidney function decline. This review shows that this problem may be solved by replacing WC with ABSI. Future research should explore whether ABSI-MetS also predicts cardiovascular events, and whether therapeutic intervention that reduces ABSI improves clinical outcomes.

## 1. Introduction

The majority of atherosclerotic diseases develop based on a constellation of metabolic disorders, with abdominal obesity as the upstream pathophysiology. This concept has been combined to define metabolic syndrome (MetS), and it has been demonstrated that MetS increases the mortality and mortality of cardiovascular disease (CVD) [1]. On the other hand, whether abdominal obesity *per se *exerts vascular toxicity independent from various metabolic disorders is controversial. Concerns exist that the current diagnostic criteria for MetS based on abdominal obesity cannot adequately predict the risk of arteriosclerosis and kidney function decline [2]. In other words, there is no clinical relevance in applying the concept of abdominal obesity as a requisite for a diagnosis of MetS.

While body mass index (BMI) is widely used as a standard index to define obesity, it is not suitable to estimate body fat mass or its location, nor can it distinguish abdominal obesity [3]. Consequently, waist circumference (WC) has emerged as a candidate for assessing abdominal obesity. In fact, WC has been reported to predict mortality risk better than BMI [4]. However, WC correlates closely with BMI, to the extent that differentiating the two as epidemiological risk factors can be difficult [5]. Therefore, a body shape index (ABSI) has been developed as a transformation of WC and is statistically independent of BMI allowing for better evaluation of the relative contribution of WC to abdominal obesity. ABSI predicts mortality better than WC and BMI [6], and has been reported to reflect obesity-related metabolic disorders well [7]. From these backgrounds, we hypothesized the inadequacy of WC as an abdominal obesity index in the diagnostic criteria of MetS. Furthermore, we verified the significance of utilizing ABSI as a candidate for the abdominal obesity index to predict vascular outcomes.

In this context, this brief review first presents the findings between systemic vascular function and chronic kidney disease (CKD), followed by an evidence-based discussion of appropriate abdominal obesity indices focusing on the prediction of vascular and kidney function. Finally, a proposal is put forward for the modification of the current MetS diagnostic criteria by utilizing ABSI.

## 2. Cardio-ankle vascular index (CAVI) as A Systemic Arterial Stiffness Parameter

The degree of progression of systemic arteriosclerosis can be assessed using the concept of arterial stiffness, and the arterial stiffness parameter is useful in the management of cardiometabolic disorders [8]. Pulse wave velocity (PWV) is the most commonly used quantitative assessment tool of arterial stiffness, although PWV changes according to changes in blood pressure (BP) at the time of measurement [9]. The BP-dependency of PWV has led to an overestimation of the role of hypertension in epidemiological studies. To overcome this problem, the CAVI has been developed [10].

CAVI is an arterial stiffness parameter that includes the entire arterial tree from the aortic valve to the tibial artery. This parameter has been theoretically and clinically established to be independent of BP at the time of measurement. This BP-independence gives CAVI the characteristics of a tool for evaluating the ventricular-arterial interaction. In other words, CAVI can evaluate vascular wall stiffness in acute response to hemodynamic changes, such as heart failure or sepsis. The value of CAVI in daily clinical practice is not limited to predicting cardiovascular (CV) events. CAVI reflects the severity of CVD risk factors including glucose intolerance, hypertension, dyslipidemia, sleep apnea, and smoking [10]. Since CAVI is a modifiable indicator that can be improved through appropriate therapeutic interventions, it is useful not only for screening CVD risk factors but also for determining the efficacy of treatment.

Vascular toxicity due to various CVD risk factors can be detected as an increase in CAVI, and a CAVI of 9.0 has been proposed to be the optimal cut-off value for predicting CVD in Asian people [10]. The prognostic value of CAVI has been well established, and increased CAVI is an independent predictor of all-cause mortality that is superior to PWV, even in the sub-population of individuals with end-stage kidney disease [11]. Notably, the prognostic value of CAVI was confirmed in a multinational setting and was present even when limited to the primary prevention group [12].

## 3. Prognostic Value of CAVI for Kidney Function Decline

CKD, which is estimated to affect approximately 10% of the world’s adult population [13], is known to share a pathophysiology closely related to systemic vascular dysfunction. Decreased kidney function is often caused by CVD risk factors including diabetes and hypertension [14]. On the other hand, CKD *per se* is known to independently enhance arteriosclerosis via accumulation of uremic toxins, increased oxidative stress and chronic inflammation, altered lipoprotein metabolism and vascular calcification [15]. Therefore, to break the vicious cycle between CKD and systemic arteriosclerosis, both should be managed simultaneously in routine clinical practice. We first examined the predictive ability of CAVI, focusing on kidney function decline as a vascular outcome.

Table 1 (Ref. [16, 17, 18, 19, 20, 21, 22, 23, 24, 25, 26, 27, 28]) summarizes the association of CAVI with kidney outcomes in cross-sectional and longitudinal studies. First, we will discuss the findings of cross-sectional studies. Six cross-sectional studies [16, 17, 18, 19, 20, 21] have shown that CAVI is independently associated with estimated glomerular filtration rate (eGFR), serum cystatin C and proteinuria. Furthermore, the association between CAVI and kidney function has been established not only biochemically, but also physiologically. The renal resistive index (RRI) is measured utilizing duplex doppler ultrasonography, and is an indicator of dynamic and structural changes in the renal vasculature. Hitsumoto has reported that CAVI was independently associated with the RRI in patients with essential hypertension [21].

**Table 1.** **Summary of the association of CAVI with kidney outcomes in cross-sectional and longitudinal studies**.

References	Country	Population	Sample size	Age (y)	Baseline CAVI	Observational period	Objective variable/Outcomes	Cut-off of CAVI	Summary
Cross-sectional study									
Kubozono* et al*. 2009 [16]	Japan	Individuals undergoing health checkups	881	52 ± 14	8.5 ± 1.3	-	eGFR, CKD stage and Proteinuria	-	Independent association of CAVI with kidney function.
Nakamura* et al*. 2009 [17]	Japan	Patients with any risks and/or CAD	206	65.3 ± 8.7	9.38 to 10.7	-	Serum cystatin C and eGFR	-	Independent association of CAVI with kidney function.
Ito* et al*. 2015 [18]	Japan	Obese patients with diabetes	468	55.3 ± 11.6	7.8 ± 1.4	-	eGFR (serum Cystatin C- or Cr-based)	-	Cystatin C-based eGFR correlated most strongly with CAVI.
Liu* et al*. 2017 [19]	China	Outpatients	656	-	-	-	Urinary albumin excretion (UAE)	-	Independent association of log UAE with CAVI.
Alizargar* et al*. 2019 [20]	Taiwan	Community individuals	164	62.64 ± 9.47	8.62 ± 1.08	-	eGFR	-	Independent association of CAVI with kidney function.
Hitsumoto 2020 [21]	Japan	Patients with hypertension	245	63 ± 13	8.7 ± 1.4	-	Renal resistive index (RRI)	9.0	Independent association of CAVI with RRI.
Longitudinal study									
Itano* et al*. 2020 [22]	Japan	Participants without CKD at the baseline	24,297	46.2 ± 13.0	7.5 ± 1.0	Mean: 3.1 y	eGFR <60, and rapid eGFR decline (≤–3/y)	8.1	Persons with CAVI ≥8.1 had a higher risk for CKD events.
Satirapoj* et al*. 2020 [23]	Thailand	Patients with high CVD risk	352	67.8 ± 10.1	63.6%: CAVI ≥9	1 y	Decrease in GFR ≥5/y	9.0	Independent association of CAVI ≥9 with a rapid declined eGFR.
Jeong* et al*. 2020 [24]	Korea	Patients above 18 years	8701	60.4 ± 11.4	8.47 ± 1.21	Median: 7 y	Doubling of serum Cr, a 50% decreased eGFR or development of ESRD	-	Higher risk of the fourth quartile for ESRD than the first quartile.
Nagayama* et al*. 2022 [25]	Japan	Urban residents without renal impairment	27,864	Median 45 (IQR 36–56)	Median 7.5 (IQR 6.9–8.2)	Mean: 3.5 y	eGFR <60	8.0	Superiority of CAVI to haPWV and CAVI0 in predicting kidney function decline.
Aiumtrakul* et al*. 2022 [26]	Thailand	Subjects with CVD risks and/or diseases	4898	-	-	5 y	eGFR decline over 40%, eGFR <15, doubling of serum Cr, dialysis initiation and death related to renal causes	-	CAVI ≥9 was an independent risk for composite kidney outcomes.
Nagayama* et al*. 2023 [27]	Japan	Urban residents without renal impairment	27,864	Median 45 (IQR 36–56)	Median 7.5 (IQR 6.9–8.2)	Mean: 3.5 y	eGFR <60	-	TG and TG/HDL-C ratio was associated with CAVI-mediated kidney function decline.
Nagayama* et al*. 2024 [28]	Japan	Urban residents without renal impairment	27,648	Median 46	-	Median: 3 y	eGFR <60	8.0 (male) 7.9 (female)	Serum uric acid was associated with CAVI-mediated kidney function decline.

CAVI, cardio-ankle vascular index; CAVI0, a variant of CAVI; eGFR, estimated glomerular filtration rate (mL/min/1.73 m^2^); CKD, chronic kidney disease; CAD, coronary artery disease; CVD risk, cardiovascular disease risk; ESRD, end-stage renal disease; haPWV, heart-ankle pulse wave velocity; IQR, inter-quartile range; Cr, creatinine; TG, triglycerides; HDL-C, high-density lipoprotein cholesterol; y, year(s); GFR, glomerular filtration rate.

Next, regarding the findings of longitudinal studies. Several longitudinal studies [22, 23, 24, 25, 26, 27, 28] have also reported the predictive ability of CAVI for kidney function decline. We have reported the superior predictive ability of CAVI compared to heart-ankle PWV (haPWV) and CAVI0, a variant of CAVI [25].

**Fig. 1.**
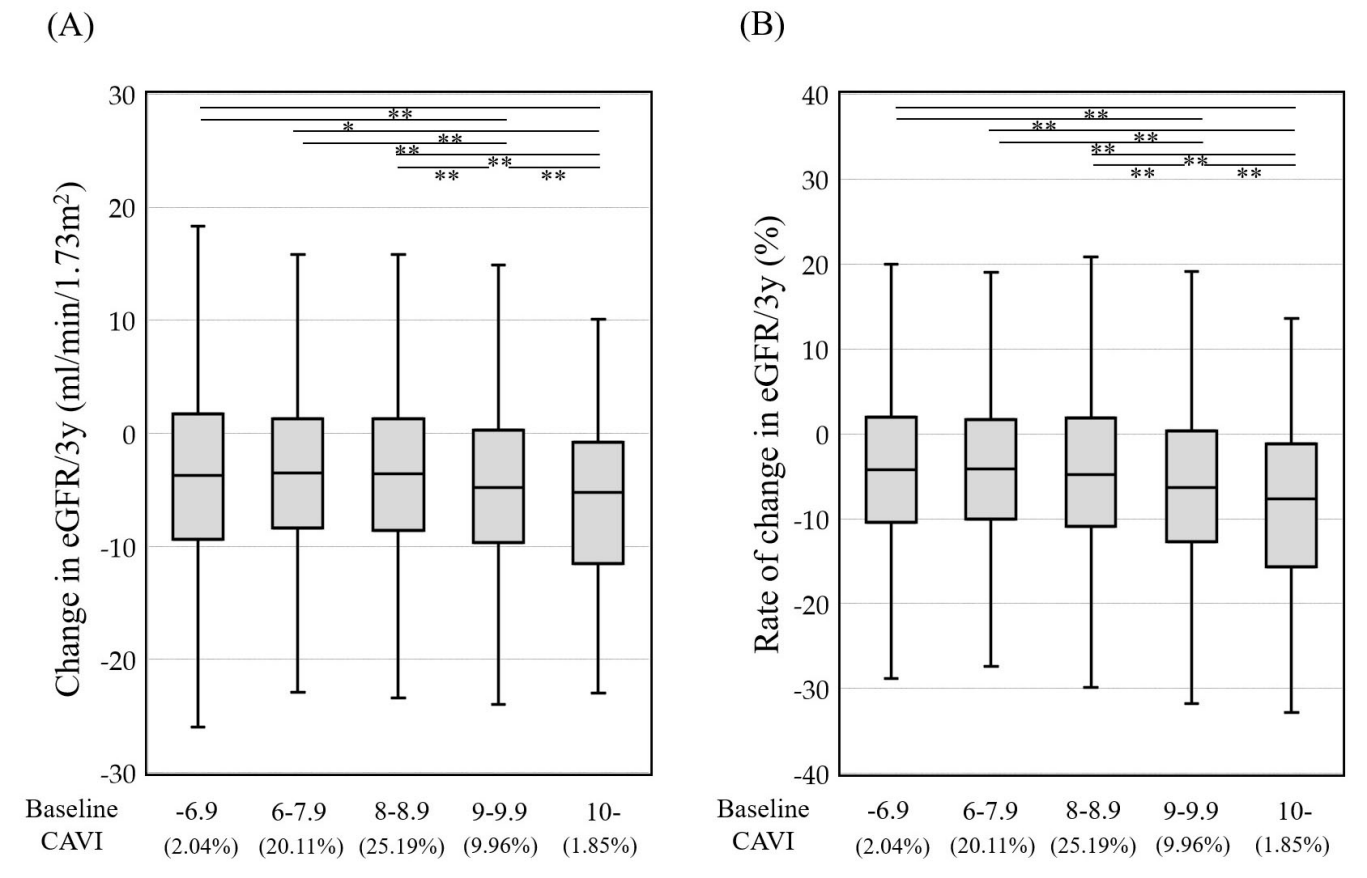
**Relationship of baseline CAVI with change (A) and (B) rate of change in kidney function over 3 consecutive years**. Participants were 11,400 Japanese urban residents with eGFR ≥60 mL/min/1.73 m^2^ who received consecutive annual health checks (median age: 48 years at baseline, male: 46.0%). *p < 0.05, **p < 0.01; Kruskal-Wallis test followed by Bonferroni test. CAVI, cardio-ankle vascular index; eGFR, estimated glomerular filtration rate (mL/min/1.73 m^2^). 3y, 3 consecutive years. Data from Nagayama D, Watanabe Y, Fujishiro K, *et al*. [28].

In general, increased CAVI precedes kidney function decline. Fig. 1 (Ref. [28]) shows the relationship of baseline CAVI with a change in kidney function over 3 consecutive years. Among Japanese individuals who underwent health screening, a baseline CAVI above 9 was associated with a greater decline in kidney function.

Increased CAVI reflects not only organic lesions in macrovessels but also microcirculatory disorders induced by inflammation and oxidative stress [10]. In addition, arterial stiffening leads to increased pressure and flow pulsatility which are transmitted downstream to the microcirculation, including the kidneys. This excessive pulsatile load damages small arteries and glomeruli in the kidney cortex [12]. Therefore, vascular aging indicated by high CAVI may lead to kidney function decline via injury in the microvasculature. As kidney dysfunction further enhances systemic oxidative stress and inflammation, it is necessary to identify the mediators of this vicious cycle.　For example, we have reported that lipid parameters, especially serum triglycerides [27], and serum uric acid levels [28] may contribute to kidney dysfunction mediated by CAVI. Although the relationships between CAVI and kidney dysfunction are restricted in the Asian population, they are common to both health screening recipients and patients with CVD risks. On the other hand, it should be noted that the above findings may not be generalizable. As some of the studies presented in this section did not exclude primary kidney disease, the endpoint of kidney function decline is not necessarily due to increased arterial stiffness. Therefore, it is possible that conventional anti-atherosclerotic treatments that reduce CAVI may not inhibit the progression of renal decline and the subsequent development of CVD.

## 4. Relationship of Abdominal Obesity with CKD

Obesity can lead to CKD through both direct and indirect pathways [29]. A systematic review and meta-analysis predict that approximately 14% of males and 25% of females develop CKD as a clinical consequence of being overweight or obese in industrialized countries [29]. Furthermore, we have reported that maximum lifetime BMI is associated with early hemodialysis initiation independent of diabetes in Japanese hemodialysis patients [30]. On the other hand, obesity-related kidney dysfunction may be reversible, as it has been reported that weight reduction therapy utilizing a formula diet results in decreased serum creatinine in type 2 diabetes patients with obesity [31].

CKD as an atherosclerotic disease may not be adequately managed by focusing on BMI alone. The significance of focusing on visceral fat accumulation to predict a decline in kidney function is controversial. However, several cohort studies have reported that visceral fat indicators were associated with the progression of CKD. Madero *et al*. [32] showed that visceral fat area (VFA) measured by computed tomography, but not BMI and WC, was independently associated with a decline of eGFR of 30% or more in the elderly participants. In addition, Kataoka *et al*. [33] revealed that the visceral-to-subcutaneous fat ratio (V/S ratio) also predicted a kidney function decline in CKD patients. Our previous study showed that weight reduction therapy reduced CAVI in individuals with obesity [10]. Notably, ΔCAVI was independently associated with ΔVFA, but not with ΔBMI. Similarly, in the aforementioned study of weight reduction therapy using formula diet [31], a decrease in serum creatinine correlated with a decrease in VFA. These findings suggest the possibility that weight reduction therapies that reduce excess visceral fat mass may be useful in attenuating vascular and/or nephrotic toxicity. Besides, we have also reported on the relationship between abdominal obesity indices and kidney dysfunction [2]. Among Japanese urban residents receiving health screenings, several abdominal obesity indices, including ABSI, waist-to-height ratio (WHtR) and conicity index, showed stronger associations with kidney dysfunction compared with BMI and WC. Namely, it may be meaningful to focus on the presence of abdominal obesity, rather than just increased BMI, in order to manage obesity-related glomerulopathy.

## 5. Criticisms of the Current MetS Concept in Terms of Vascular Outcomes

The individual components of MetS; namely, central obesity, insulin resistance, dyslipidemia and hypertension, are all independent risk factors of atherosclerosis. The clustering of these risk factors in MetS significantly elevates the risk of developing CVD [34]. In addition, MetS is associated with CKD and microalbuminuria in both cross-sectional and longitudinal studies [35]. On the other hand, several criticisms of the MetS concept have emerged. As an example, Reaven contended that WC as an indicator of abdominal obesity is not necessarily related to insulin resistance [36]. This implies that there is no clinical relevance in applying the concept of abdominal obesity as a requisite for the diagnosis of MetS. In addition, the study of Ming *et al*. [37] confirmed an association between MetS and CKD, but elevated WC did not show significantly increased odds of CKD when adjusted for all components of MetS. Similarly, the REGARDS study [38], a longitudinal cohort study of community residents, revealed that WC was not associated with end-stage kidney disease after adjustment for obesity-related comorbidities, eGFR and urinary albumin/creatinine ratio. Taken together, the clinical utility of a diagnosis of MetS based on elevated WC is unclear, and treatment of all CVD risk factors in the individual may suffice. Alternatively, it may be preferable to utilize an anthropometric index that reflects vascular toxicity as an element of MetS, independent of BMI and WC.

## 6. Obesity Paradox Regarding Vascular Function

Flow-mediated dilation, the standard tool used to assess vascular endothelial function, has been known to correlate positively with BMI in healthy young adults [39]. This finding corroborates reports of an inverse relationship between CAVI and BMI in the general Japanese and Chinese population [10, 40]. In other words, increased BMI is associated with improved vascular function, despite being a risk factor for metabolic disorders. BMI is therefore speculated to primarily reflect a body composition with a vascular protective effect (such as subcutaneous fat and/or skeletal muscle). This finding is consistent with the “obesity paradox” phenomenon, an observation that obesity may contribute to improved survival in patients with CVD [41]. Similarly, in support of this paradoxical finding, it has been reported that when accompanied by obesity, the risk of all-cause or CVD mortality in MetS patients may be reduced to the same level as in non-MetS individuals [42].

Current MetS diagnostic criteria adopt WC as a simple indicator of visceral fat accumulation, as described above. However, since WC correlates strongly with BMI, the two are almost epidemiologically identical [43]. Moreover, Sugiura *et al*. [44] reported that not only BMI, but also WC, correlated inversely with CAVI in Japanese workers, while visceral fat area correlated positively. Reaven [36] emphasized that there are many non-MetS patients who are clearly at higher risk of CVD than MetS patients with high WC. In other words, the current MetS criteria, which requires high WC, also runs the risk of identifying individuals who benefit from the vasoprotective effects of increased subcutaneous fat and/or skeletal muscle. This speculation is also supported by the fact that, in the Advanced Approach to Atherosclerosis study [45], a multicenter cross-sectional study in Europe, individuals with high WC showed relatively low CAVI, despite having the same degree of metabolic disorders. Based on the above, the suitable abdominal obesity index as a surrogate marker of visceral fat accumulation that exerts vascular toxicity should be independent of the “obesity paradox”. ABSI, one of the abdominal obesity indices mentioned above, was designed to be minimally associated with BMI, and is calculated by dividing WC by an allometric regression of weight and height. In other words, the ABSI is an index for quantifying the transverse diameter of the body without the influence of the “obesity paradox”.

## 7. ABSI as a Novel Anthropometric Index Reflecting Vascular Health

Recently, a number of anthropometric indices have been developed to quantify the degree of abdominal obesity more precisely than WC. Furthermore, these indices have been shown to have better predictive ability for CVD outcomes. For example, in the Norwegian Nord-Trøndelag Health Study 2, in which 61,016 participants were followed for an average of 17.7 years, ABSI and WHtR, were both more strongly associated with CV mortality than the established indices including BMI, WC and waist-to-hip ratio [46]. The association between ABSI and CVD mortality did not alter even in a sensitivity analysis excluding participants with high risk for CVD mortality at baseline (i.e., known CVD, diabetes and current smokers). Similarly, ABSI outperformed BMI and WC in predicting CVD and all-cause mortality in meta-analyses [46, 47, 48]. Furthermore, findings showing an association between abdominal obesity indices and potential CVD risk factors were also confirmed. Among the 3140 participants extracted from the National Health and Nutrition Examination Survey (NHANES) 2013–2014, increased ABSI was closely related to a high risk of abdominal aortic calcification (AAC), and the discriminative power of ABSI for AAC was significantly higher than that of BMI, WC, and WHtR [49]. Besides, a prospective study of 1718 Korean general residents found that WHtR, unlike BMI and waist-to-hip ratio, was independently associated with new-onset hypertension [50]. These findings suggest that some form of abdominal obesity index, rather than WC, should be utilized to effectively identify atherosclerotic disease.

**Fig. 2.**
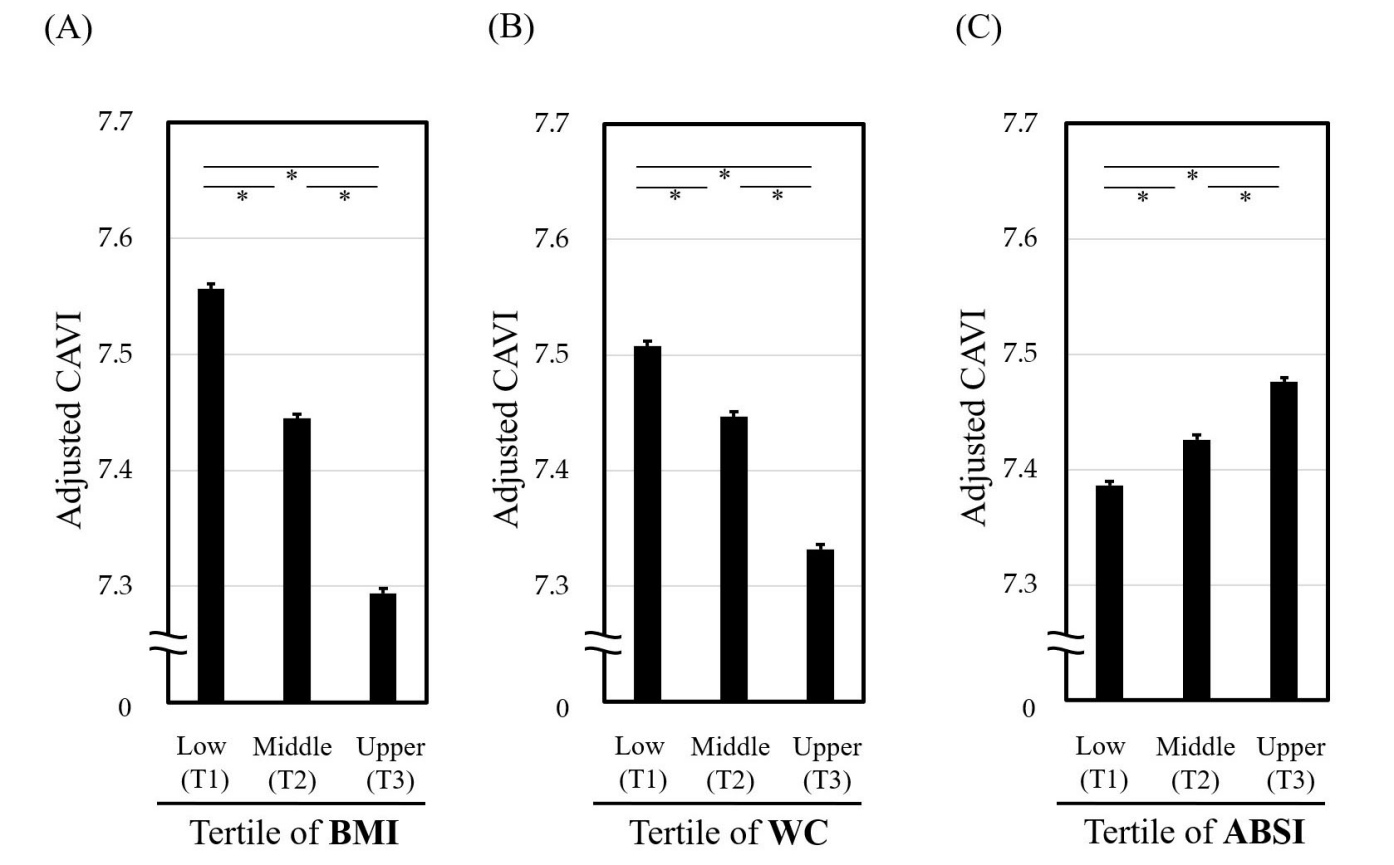
**Relationship of CAVI with tertiles of (A) BMI, (B) WC and (C) ABSI**. *p < 0.001, ANOVA followed by post-hoc Bonferroni test. CAVI was adjusted by gender, age, and SBP. A total of 62,514 Japanese urban residents who received health checks (median age: 42 years, male: 41.6%) were studied. CAVI, cardio-ankle vascular index; BMI, body mass index; WC, waist circumference; ABSI, a body shape index; ANOVA, analysis of variance; SBP, systolic blood pressure. Data from Nagayama D, Watanabe Y, Yamaguchi T,* et al*. [43].

We previously examined the relationship of vascular function with various abdominal obesity indices, using CAVI. In a cross-sectional study of Japanese subjects undergoing health screening, we examined the association of CAVI with abdominal obesity indices comprising ABSI, WC, WHtR, WC/BMI ratio and the conicity index [2]. In this study, ABSI showed the highest discriminatory power for CAVI above 9.0 (the cutoff value for coronary artery disease) compared to the other indices. In addition, stratified analyses dividing each index into tertiles showed that BMI and WC correlated negatively with confounders-adjusted CAVI (Fig. 2A,B, Ref. [43]), whereas ABSI correlated positively (Fig. 2C). Furthermore, ABSI of 0.080 was the cutoff value corresponding to a CAVI of 9.0 in both sexes. These findings suggest that high ABSI, defined for convenience as 0.080 or higher, may be the most suitable marker of abdominal obesity. We thus propose replacing high WC with high ABSI in MetS diagnosis criteria to refine risk stratification, as shown in Table 2.

**Table 2.** **Proposal to replace high WC by high ABSI as the criterion of visceral obesity in MetS diagnosis**.

	Japanese criteria	IDF criteria for Asians	NCEP-ATPIII criteria
Definition of MetS	(1) + any two or more of (2) to (4)	(1) + any two or more of (2) to (5)	Three or more of (1) to (5)
**Components**			
Visceral obesity	WC ≥85 cm (males)	WC ≥90 cm (males)	WC ≥102 cm (males)
	≥90 cm (females)	≥80 cm (females)	≥88 cm (females)
Proposal to replace high WC by high ABSI (≥0.080, both sexes) as the criterion of visceral obesity			
Hypertension	(2) SBP ≥130 mmHg and/or	(2) SBP ≥130 mmHg and/or	(2) SBP ≥130 mmHg and/or
	DBP ≥85 mmHg	DBP ≥85 mmHg	DBP ≥85 mmHg
Hyperglycemia	(3) FPG ≥110 mg/dL (6.11 mmol/L)	(3) FPG ≥100 mmol/L (5.55 mmol/L)	(3) FPG ≥100 mg/dL (5.55 mmol/L)
Dyslipidemia	(4) TG ≥150 mg/dL (1.69 mmol/L)	(4) TG ≥150 mg/dL (1.69 mmol/L)	(4) TG ≥150 mg/dL (1.69 mmol/L)
	and/or		
	HDL-C <40 mg/dL (1.03 mmol/L)		
		(5) HDL-C <40 mg/dL (1.03 mmol/L, males)	(5) HDL-C <40 mg/dL (1.03 mmol/L, males)
		<50 mg/dL (1.29 mmol/L, females)	<50 mg/dL (1.29 mmol/L, females)

WC, weight circumference; ABSI, a body shape index; SBP, systolic blood pressure; DBP, diastolic blood pressure; FPG, fasting plasma glucose; HDL-C, high-density lipoprotein-cholesterol; TG, triglyceride; MetS, metabolic syndrome; IDF, International Diabetes Federation; NCEP-ATPIII, National Cholesterol Education Program-Adult Treatment Panel III. Adapted from Nagayama D, Sugiura T, Choi SY, *et al*. [2].

All three representative diagnostic criteria for MetS (Japanese, IDF, and NCEP-ATPIII) use WC as the indicator of abdominal obesity. However, their cutoff values differ from each other. We propose to change the criteria for abdominal obesity in all three diagnostic criteria, replacing high WC with ABSI ≥0.080 for both men and women. Furthermore, we examined whether using ABSI-based MetS criteria (ABSI-MetS) has superior clinical significance than the conventional WC-based MetS criteria (WC-MetS).

## 8. Clinical Significance of Replacing High WC with High ABSI in MetS Diagnostic Criteria

**Fig. 3.**
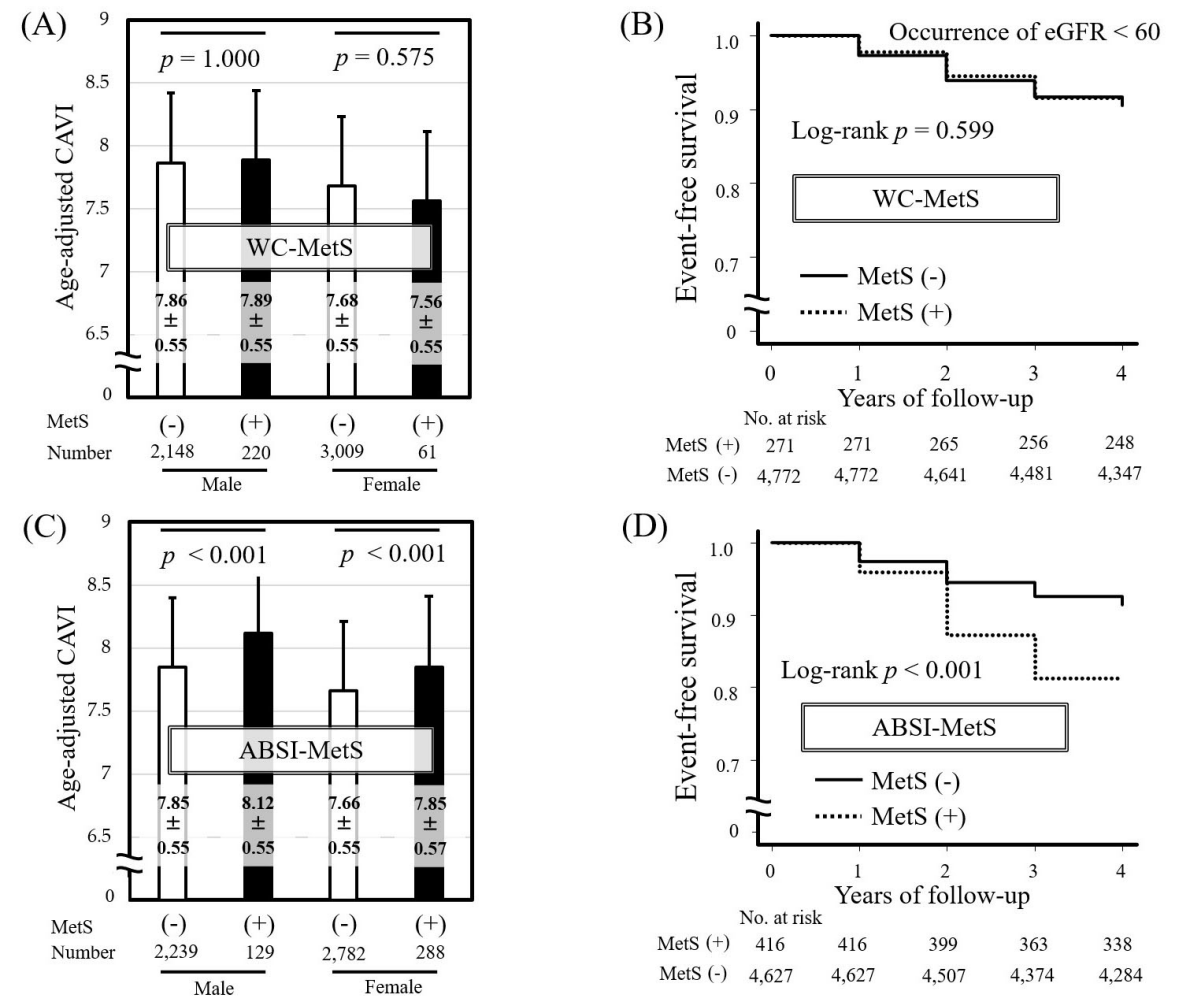
**Comparison of the predictive ability of WC-MetS and ABSI-MetS for CAVI and kidney function decline**. Comparison of age-adjusted CAVI in MetS (+) vs. MetS (-) individuals diagnosed by Japanese MetS criteria using (A) WC (WC-MetS) or (B) ABSI (ABSI-MetS). Data are presented as mean ± standard deviation and analyzed by one-way analysis of covariance with the covariate set to age followed by Bonferroni multiple comparison test. Kaplan-Meier curves for the rate of renal function decline in MetS (+) and MetS (–) individuals diagnosed by Japanese MetS criteria using (C) WC-MetS or (D) ABSI-MetS. Kidney function decline was defined as the occurrence of eGFR <60 mL/min/1.73 m^2^. A total of 5438 Japanese urban residents (median age 48 years at baseline, male 43.5%) without kidney impairment who participated in a public health screening program for 4 consecutive years were studied. WC, waist circumference; ABSI, a body shape index; MetS, metabolic syndrome; CAVI, cardio-ankle vascular index; eGFR, estimated glomerular filtration rate (mL/min/1.73 m^2^). Data from Nagayama D, Sugiura T, Choi SY, *et al*. [2].

We examined the clinical significance of ABSI-MetS in 5438 Japanese urban residents (median age 48 years) who participated in a health screening program for four consecutive years, as shown in Fig. 3 (Ref. [2]). MetS was essentially diagnosed using the Japanese criteria.

Individuals diagnosed with MetS using ABSI-MetS had significantly higher age-adjusted CAVI compared with non-MetS individuals (Fig. 3C), whereas no significant difference in age-adjusted CAVI was found between MetS and non-MetS individuals diagnosed using WC-MetS (Fig. 3A). The finding that individuals with MetS diagnosed using WC-MetS do not necessarily show high CAVI is consistent with the results of a large European multicenter study [45]. Similarly, replacing high WC with high ABSI in MetS diagnostic criteria has been shown to be useful for identifying individuals with relatively high CAVI in studies of Japanese workers [51] and in the general Korean population [46]. Furthermore, in our study, Kaplan-Meier analysis of new-onset kidney function decline (eGFR <60 mL/min/1.73 m^2^) showed a significant difference in the incidence of kidney function decline between Met and non-Met individuals diagnosed using ABSI-MetS (Fig. 3D), but no significant difference between the two groups when using WC-MetS (Fig. 3B). The ability of ABSI-MetS to predict kidney function decline remained after adjustment for confounders including age, proteinuria, and treatment for metabolic disorders in a Cox-proportional hazard analysis. In other words, replacing high WC with high ABSI in the MetS diagnostic criteria more efficiently identified subjects at risk of kidney function decline and systemic arterial stiffening.

## 9. Concern and Prospect Regarding ABSI

Unlike WC, ABSI can precisely assess the vascular toxicity induced by visceral fat accumulation by minimizing the vasoprotective body composition influence reflected in BMI (i.e., the obesity paradox). However,　it is unclear what therapeutic interventions can effectively reduce ABSI, and it is also not known whether improved ABSI contributes to a reduction in clinical outcomes. Moreover, there is a limitation regarding ABSI in that it is inferior to existing abdominal obesity indices in identifying abdominal obesity-related metabolic disorders [52]. To substantiate this, ABSI has also been reported to have a relatively weak association with visceral fat areas evaluated by computed tomography [53].

As an explanation for this unsolved concern mentioned above, we speculate a possibility that high ABSI does not necessarily reflect excess visceral fat accumulation. We have reported that increased ABSI reflects abdominal bulging beyond that expected for a given BMI, or a geometric change from cylindricity to conicity [2]. Hence, reduced skeletal muscle mass relative to WC may also result in higher ABSI. This consideration is consistent with a previous report that stated high ABSI reflects the risk of sarcopenia, including decreased hand grip strength [54]. Recently, sarcopenia *per se* has been associated with vascular dysfunction [55] and kidney function decline [56]. In the future, the detailed relationship of ABSI with body composition needs to be clarified, including skeletal muscle mass. If this is achieved, MetS diagnosis utilizing ABSI has the potential to identify new pathophysiological conditions beyond visceral fat accumulation.

## 10. Conclusions

The present review illustrates that the use of ABSI in MetS criteria can detect individuals with arterial stiffening, which may lead to efficient stratification of the risk of kidney function decline. Future research should examine the following clinical questions: (1) whether MetS diagnosis utilizing high ABSI also predicts CVD morbidity and mortality; and (2) whether a therapeutic intervention that reduces ABSI is useful for improving clinical outcomes, including metabolic disorders and atherosclerotic diseases.
